# Development and implementation of a distributed data network between an academic institution and state health departments to investigate variation in time to HIV viral suppression in the Deep South

**DOI:** 10.1186/s12889-023-15924-0

**Published:** 2023-05-24

**Authors:** John R. Bassler, Izza Cagle, Danita Crear, Emma S. Kay, Dustin M. Long, Michael J. Mugavero, Ariann F. Nassel, Lauren Ostrenga, Mariel Parman, Summer Preg, Xueyuan Wang, D. Scott Batey, Aadia Rana, Emily B. Levitan

**Affiliations:** 1 Department of Biostatistics, University of Alabama at Birmingham, Birmingham, AL USA; 2 Office of HIV Prevention and Care, Alabama Department of Public Health, Montgomery, AL USA; 3 Vaccine-Preventable Diseases and Immunization Program, Tennessee Department of Health, Union City, TN USA; 4 Magic City Research Institute, Birmingham AIDS Outreach, Birmingham, AL USA; 5 Department of Medicine, University of Alabama at Birmingham, Birmingham, AL USA; 6 University of Alabama at Birmingham, Lister Hill Center for Health Policy, Birmingham, AL USA; 7 Louisiana Department of Health, New Orleans, LA USA; 8 STD/HIV Office, Mississippi State Department of Health, Jackson, MS USA; 9 School of Social Work, Tulane University, New Orleans, LA USA; 10 Department of Epidemiology, University of Alabama at Birmingham, Birmingham, AL USA

**Keywords:** HIV, Public health, Surveillance, Community partnerships, Distributed data network

## Abstract

**Background:**

Achieving early and sustained viral suppression (VS) following diagnosis of HIV infection is critical to improving outcomes for persons with HIV (PWH). The Deep South of the United States (US) is a region that is disproportionately impacted by the domestic HIV epidemic. Time to VS, defined as time from diagnosis to initial VS, is substantially longer in the South than other regions of the US. We describe the development and implementation of a distributed data network between an academic institution and state health departments to investigate variation in time to VS in the Deep South.

**Methods:**

Representatives of state health departments, the Centers for Disease Control and Prevention (CDC), and the academic partner met to establish core objectives and procedures at the beginning of the project. Importantly, this project used the CDC-developed Enhanced HIV/AIDS Reporting System (eHARS) through a distributed data network model that maintained the confidentiality and integrity of the data. Software programs to build datasets and calculate time to VS were written by the academic partner and shared with each public health partner. To develop spatial elements of the eHARS data, health departments geocoded residential addresses of each newly diagnosed individual in eHARS between 2012–2019, supported by the academic partner. Health departments conducted all analyses within their own systems. Aggregate results were combined across states using meta-analysis techniques. Additionally, we created a synthetic eHARS data set for code development and testing.

**Results:**

The collaborative structure and distributed data network have allowed us to refine the study questions and analytic plans to conduct investigations into variation in time to VS for both research and public health practice. Additionally, a synthetic eHARS data set has been created and is publicly available for researchers and public health practitioners.

**Conclusions:**

These efforts have leveraged the practice expertise and surveillance data within state health departments and the analytic and methodologic expertise of the academic partner. This study could serve as an illustrative example of effective collaboration between academic institutions and public health agencies and provides resources to facilitate future use of the US HIV surveillance system for research and public health practice.

## Introduction

Achieving early and sustained viral suppression (VS) following diagnosis of HIV infection is critical to improving outcomes for persons with HIV (PWH) and reducing transmission [1–4]. This is particularly germane to the Deep South of the United States (US), a region that is disproportionately impacted by the domestic HIV epidemic [5]. More than half of new HIV diagnoses in the US have been in Southern states, where there is additionally a disproportionate impact on racial/ethnic minorities, and a high proportion of PWH in the South are undiagnosed [6]. Additionally, new diagnoses among individuals living outside of urban areas are more common in the South than in other areas of the country [6].

Prior work in Alabama showed wide variation in the time to VS across public health regions [7]. Socio-contextual factors influence the well-being of a person and include factors such as discrimination, neighborhood violence, and access to healthcare, food, and transportation [8]. Where PWH reside may be as critical, if not more so, than individual-level factors in terms of the ability to connect to an HIV provider and stay in care. A deeper understanding of these factors driving geographic variability of VS in the Deep South can guide the development of evidence-informed public health approaches to achieve timely individual and population viral control, aligning with the key principles of the Ending the HIV Epidemic (EHE) initiative.

Our current National Institute of Allergy and Infectious Diseases-funded study, Road to Zero, is the result of collaboration between our academic institution, the University of Alabama at Birmingham (UAB), and the state public health departments in Alabama (ADPH), Mississippi (MDPH), and Louisiana (LDH). In line with the goals of the EHE, this study aims to quantitatively and qualitatively explore the role of individual and contextual characteristics in time to VS, using HIV surveillance data to characterize geographic variability in the time from HIV diagnosis to VS, identifying individual- and community-level determinants of time to VS using geospatial methods, and contextualizing these finding with qualitative interviews among PWH, HIV treatment and social services providers, and community stakeholders.

Public health agencies collect detailed and sensitive individual level data about PWH and have on the ground experience and expertise. Years of experience coordinating public health efforts in the communities they serve, identifying trusted community partners, and practical knowledge of the concerns and obstacles that PWH face, while not always quantifiable, is immensely valuable. Yet, health departments do not typically have access to the advanced statistical programming needed to perform complex analyses that reveal local epidemiological trends and help foster data-driven efforts. Academic investigators can bring technical and methodologic expertise in research design and analytics, both quantitative and qualitative, and can help troubleshoot any potential barriers to collaborative efforts between academic institutions and health departments [9].

The purpose of this report is to describe collaborative processes, structure, and tools developed as part of Road to Zero which leveraged the strengths of the public health agencies and the academic partner.

## Methods

The Road to Zero project was funded by a research project grant (R01AI142690) from the National Institute of Allergy and Infectious Diseases, part of the US National Institutes of Health. The academic partner, UAB, served as the primary grantee and coordinating center, with subcontracts to the state public health departments to support the time and effort of public health department employees, software, and associated expenses such as travel to study meetings.

### Collaborative processes (Fig. 1)

**Fig. 1:**
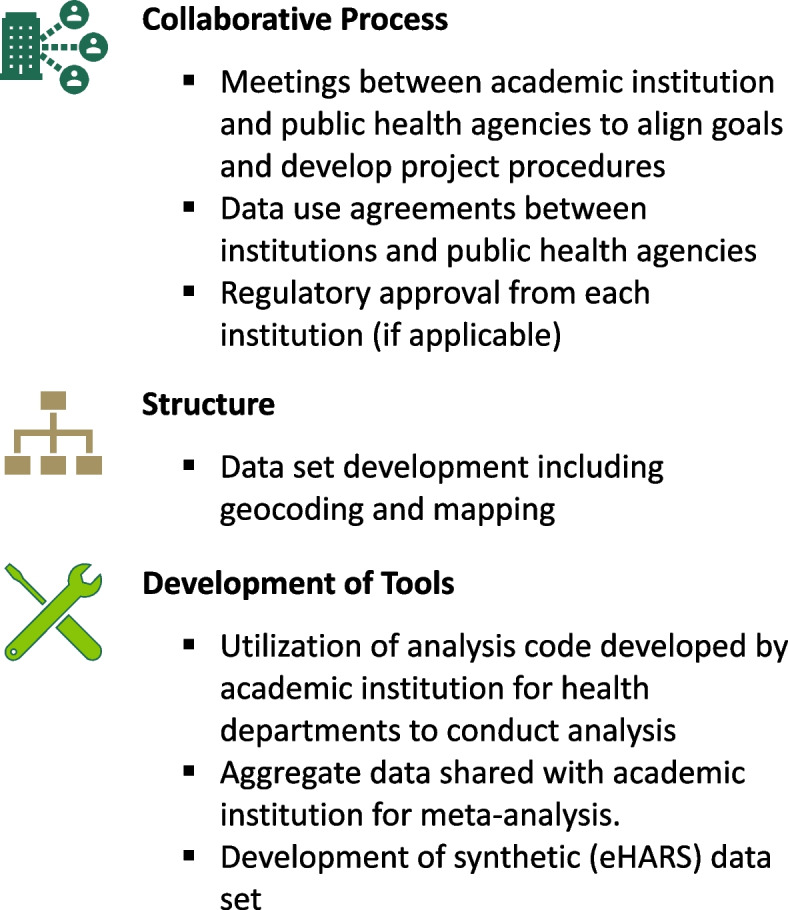
Checklist of activities to develop and implement distributed data network between academic institution and public health agencies

**Fig. 2:**
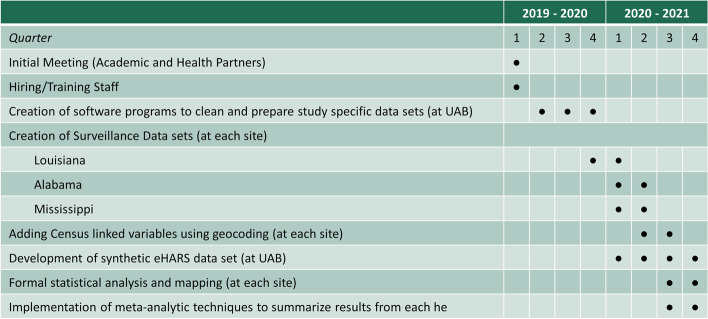
Road to Zero study timeline of project tasks and significant events

The Road to Zero study began with a two-day meeting including representatives from each of the health departments, the Centers for Disease Control and Prevention (CDC), and the academic partner. At this meeting, the partners became acquainted with each other, clarified the goals of the study, reviewed and revised the proposed quantitative methods, and identified shared priorities. To ensure the integrity of the data and appropriateness of our analytic design, members from our team and each health department identified key variables, established core objectives and outcome measures, and determined a timeline for completion (Fig. 2). This included discussion of how HIV surveillance was conducted in each state and what data were routinely collected for each person newly diagnosed with HIV. Regulatory procedures to maintain confidentiality and integrity of sensitive personal data about PWH were developed including data use agreements; and, ultimately, study methods and design were approved by the UAB Institutional Review Board (IRB) and the IRBs of each state health department. This included agreed upon plans to conduct data analysis in parallel at each state health department, and for only non-identifiable, population-level summary statistics to be shared with our academic investigators.

### Distributed data model

Detailed and accessible data on the identity, geographic location, and clinical and demographic characteristics of PWH are needed to evaluate associations with time to VS. Public health agencies collect and maintain the CDC-developed Enhanced HIV/AIDS Reporting System (eHARS) which contains this information [5]. eHARS is a standardized document-based surveillance database used by state health departments to collect and manage case reports, lab reports, and other documentation on PWH and subsequently report to the CDC. A potential barrier to partnerships between public health agencies and academic partners has been the valid concern over the privacy of PWH. Maintaining the confidentiality of the data is a critical component to our ability to work with our public health partners.

**Fig. 3:**
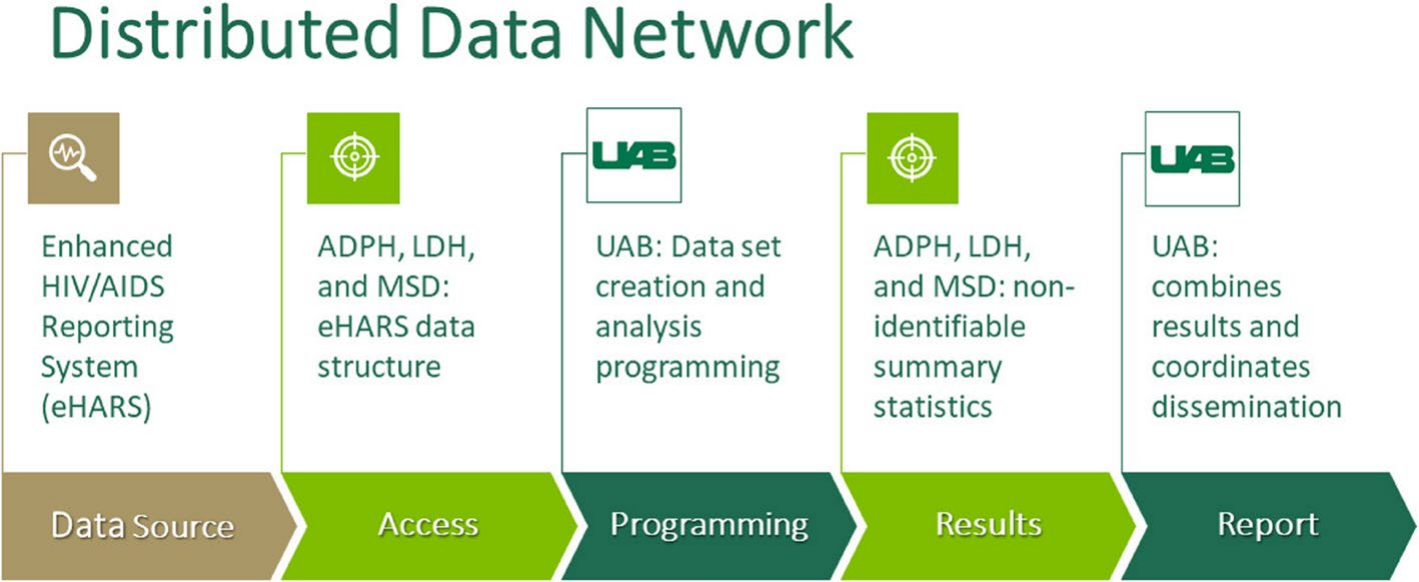
Distributed data network detailing the protection and analyses of sensitive health information

To mitigate concerns about security and confidentiality of HIV surveillance data, we used a distributed data model, or distributed data network (Fig. 3), to avoid sharing personally identifiable data collected under each public health surveillance authority [10, 11]. In this model, the health departments conduct all analyses within their own systems, with support from the academic partners, with summary results shared and pooled. As eHARS is an HIV surveillance database and access to identifiable information is strictly limited to health departments, this systematic approach allows for the utilization of eHARS data without compromising privacy.

Next steps in our process hinged on the foundation that was built in these initial meetings. Developing a positive relationship with our public health department partners was more than just adherence to protocols, but fostering a rapport based on mutual respect based on the experience and dedication brought to the project by all team members. For the data to be utilized, SAS (version 9.4 of the SAS System for Windows, Cary, NC, USA) software programs to clean and build a data set for our specific study analysis were written at UAB and then shared with each public health partner.

### Geocoding and mapping

The residential addresses of each newly diagnosed individual in eHARS between 2012–2019 was geocoded by the HIV surveillance department of each state health department, with support from the academic partner, using Geographic Information System (GIS) software (ArcGIS Desktop and Esri’s StreetMap premium street network dataset). The geocoding process converts each address into geographic coordinates and maps the corresponding point location. Once mapped, each residential address point location was assigned the geoidentifier of the individual census tract that contains it, and the number of residential locations within the boundaries of each US Census tract was counted. This enabled each health department to link Census socio-economic data at the census tract level, which is often used as a proxy for an individual’s neighborhood. Once each individual residential location was assigned a Census tract geoidentifier, the original address and mapped location were removed from analysis datasets to protect address information, which is considered personal health information.

The use of GIS in public health and disease surveillance has grown in recent years as a means of better understanding how the context of where people live, learn, and work affects their health outcomes [12]. Health departments have been using GIS to varying degrees throughout the country to conduct this type of analysis. Geocoding of eHARS data using a CDC-provided software program has been recommended by the CDC since 2017, yet, due to personnel and budgetary constraints, not all health department partners had been able to implement routine geocoding prior to the Road to Zero project.

### Synthetic data set

To maintain the ability to troubleshoot statistical analysis programs, but minimize the time needed from the health department partners, we created a synthetic data set that mirrors the eHARS data, including exact formats for all variables, realistic address data, and simulated dates for birth, HIV diagnosis, AIDS diagnosis, and death. The date variables were given careful attention to ensure that dates occurred in a natural progression and varied in time between event dates. Additionally, patient visit dates and viral load data were also simulated so that time to VS could be estimated. Although some of the assumptions included in the simulation are study-specific and most attention was given to simulating realistic data for variables needed for this project, the process could be expanded to include all eHARS variables or a different study design. This synthetic dataset has been used in our research to troubleshoot SAS code to create reports, figures, and test analysis. Although the involvement of health department partners is critical to overall success of the analysis, the synthetic data set prevents using our partners’ time to debug code, avoids using sensitive data about PWH when not necessary, and creates a platform to evaluate the viability of future hypotheses to be tested using eHARS data.

## Results

The initial collaborative meeting resulted in refinement of the study plan. For example, based on publicly available documentation of eHARS, the academic collaborators had initially selected for analysis the individual-level variables age at diagnosis, HIV transmission risk factor, race/ethnicity, current gender, presenting CD4 count, stage 3 HIV (AIDS) at diagnosis, insurance type, diagnosis facility type, education, and marital status. However, the state health department partners indicated that the insurance type, education, and marital status fields were rarely populated. There were some differences across states in how and under what circumstances the current gender field was completed, and the team decided that sex assigned at birth, while conceptually different, would be a more reliable variable. Additionally, diagnosis facility type information would require substantial data cleaning and harmonization across states, so analysis of this variable was deferred to a later time after other high priority analyses occurred.

**Fig. 4:**
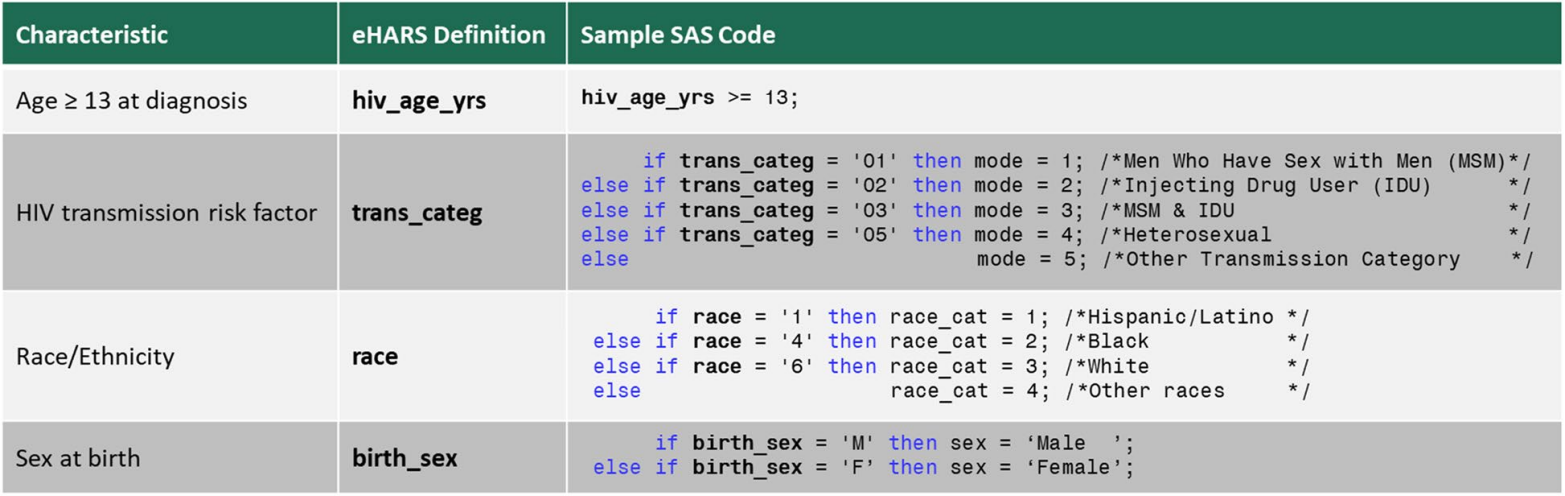
Study characteristics matched with eHARS specific variables and SAS programs written by academic investigators, with the support of public health partners

Even with the extensive groundwork to design the study and build the team, writing SAS programs without being able to test them required extensive trial and error. Through experimentation and making necessary adjustments, iterations of code were improved until an error free program could produce the desired output at LDH, which agreed to be the pilot site for analysis. Issues confronted and worked through included identifying appropriate data formats for eHARS variables, navigating differences between SAS variable types, designing output to be automatically stored in local folders, handling missing data, creating new variables, using specific SAS functions to store summary data for meta-analysis, and correcting any syntax errors. An illustration of programming study specific characteristics to prepare eHARS data for analysis is provided in Fig. 4.

The process of preparing the data for analysis was challenging. The time to VS outcome is not collected or calculated within eHARS. Time to VS from diagnosis was determined by calculating the time from documented HIV diagnosis to the first date that recorded a viral load result having less than 200 copies of HIV per milliliter of blood. Specific accommodations to program a robust estimate of time to VS include the following: individuals were censored if they had died or moved out of the state or if viral suppression was not achieved during the study period, incomplete dates for either HIV diagnosis or viral load sample were included if the year and month were available and the first day of the month was added to complete the date (i.e., January 2015 was converted to January 1^st^, 2015), and all dates were checked for chronological correctness to ensure that all time calculations were feasible. Additionally, PWH whose first viral load was reported as less than 200 copies per milliliter were excluded because these individuals are likely to be re-diagnosed instead of newly identified.

The three health department partners had small but growing GIS capacity at the start of the Road to Zero study. Identifying who administrated and organized GIS within and for each health department was often challenging. This illustrates how new technology can be siloed or contained within a single area/department within an organization and may not be available to all groups who could benefit from the technology. At the beginning of our collaboration, the HIV surveillance divisions at the state health departments had limited training and access to GIS software. One of the goals with the use of GIS for geocoding and mapping was to assist each HIV surveillance division with the growth of their capacity to use GIS through individual training regarding geocoding, spatial analysis, and mapping. We initially planned in-person multiday working sessions at each health department. However, due to the COVID-19 pandemic, many of these sessions had to be moved to video conference. The implementation of GIS is still relatively a niche service in many health departments; by providing expertise and assistance in obtaining software and data packages, we were able to avoid many common issues that starting this process can have.

Because of the complexity of eHARS and limitations on the academic partners’ ability to interact with it, developing the synthetic dataset also proved more time consuming than initially anticipated. However, with the collaboration of the state health departments, particularly LDH, a functioning synthetic version of eHARS that does not contain any individual identifying information was created and the code used to create it uploaded to a repository. In total, publicly available code in this repository include simulation of an eHARS data set (synthetic data set), data management to clean the synthetic data set and prepare the outcome Time to VS for analysis, formats and macros used to analyze the data, and an example of a basic descriptive analysis [13].

Triangulation of the findings of the quantitative analyses developed through the processes described in this report and qualitative work contextualizing statistical analysis is currently underway. The results of these analyses will be described in forthcoming publications.

## Discussion and lessons learned

### Health department perspective

Public health departments are tasked with navigating the challenges of being a government entity while serving the needs of vulnerable people in public health. The capacity and resources needed to fulfill these requirements can leave little time or funds to expand upon more specialized or analytically complex public health endeavors. In sharing the results of our analysis conducted using Louisiana-specific eHARS data, we were able to highlight the progress being made in time to VS, as well as geographic areas and demographic groups in which we need to focus our efforts to end the epidemic. These results were ultimately included in the aggregate analysis that serves the purpose of our specific research aims. A secondary result of this innovative university/public health department collaboration is that all three state health departments now have the expanded their infrastructure and expertise needed to conduct their own detailed analyses to inform targeted, evidence-based interventions.

### Academic partner perspective

Working closely with public health agencies greatly enriched our ability to conduct investigations into time to VS. The most obvious benefit was the ability to use HIV surveillance data for research purposes. As importantly, the health department partners provided invaluable local context and insight into the populations they served, the data, and the data collection methods.

### COVID-19

The pandemic created challenges for people of all professions, and this fact is particularly true for each of the public health partners who were charged with responding to the emergency on top of their usual responsibilities. Temporarily shifting roles to assist in COVID related needs, working from home, and training new staff are just some of the obstacles our partners confronted, and our team was still able to make progress in our research during this time. This can be directly attributed to the structure of the distributed data network. The programs that were needed to be run by our health department partners were written and tested prior to being run at each site. Virtual meetings to run the programs would typically last 30–60 min and occur on an ‘as-needed’ basis, occurring once a week for most of the study period. The time and resources of each of our partners were always priorities and were fully considered before any meeting was scheduled.

## Conclusion

With the implementation of this distributed data network, we were able to access data that, under traditional circumstances, is not publicly available, and we would not have the resources to collect ourselves. While academic-public health agency partnerships are not unprecedented and have been successful elsewhere, our efforts have strengthened collaborations in the Deep South. This work serves as a platform for future research among the current collaborators and a potential model for additional partnerships in the US context. We believe that this process is under-utilized, as the success of our partnership has yielded study infrastructure and analysis capabilities that would not be feasible without the existence of this relationship. Additionally, in an effort to foster future academic institution/health department relationships, a synthetic eHARS data set and code to estimate time to VS have been made publicly available to reduce the front-end engagement that could be a barrier for health departments to participate. Ultimately, this research and methods used to accomplish our goals directly align with the vision of EHE initiative. Most importantly, our research is a testament to what can be accomplished by establishing synergetic relationships among academic and public health partners.

## Data Availability

To foster future collaborations between public health departments and researchers, the SAS code to create the synthetic eHARS data set; and specifically, SAS code that calculates time to viral suppression using the simulated data is freely available. The programs and synthetic datasets generated during the current study are available in the repository.https://github.com/JRBassler/eHARS-Simulation [13].
